# Intensive care medicine in europe: the state of the training art

**DOI:** 10.1186/2197-425X-3-S1-A859

**Published:** 2015-10-01

**Authors:** A Wong, K Donadello, S Thiessen, N Aissaoui, B Bollen Pinto, G De Pascale, M Hilty, K Lane, M Mendoza, P Schellongowski, SJ Shepherd, F Weidanz, B Weiss, J Werner, L Prisco

**Affiliations:** NEXT Committee, European Society of Intensive Care Medicine, Brussels, Belgium

## Introduction

In many European Union (EU) countries, Intensive Care Medicine (ICM) remains a 'sub-specialty'. Many clinicians/ researchers envision the figure of dedicated intensivists who practice exclusively ICM. Distinct local differences exist in the minimum knowledge, skills, duration of training and non-technical behaviours and this may result in subsequent heterogeneous working conditions.

## Objectives

To characterise EU training patterns and the perception on the quality of education and working conditions.

## Methods

A web-based multi-question survey (SurveyMonkey^®^) was prepared and delivered via email to all ESICM members, so as to be received by all related ICM trainees and young specialists. Descriptive questions and a 5-point Likert scale were used. The survey was run for one year, thereafter the collected data were anonymously analyzed (Microsoft Excel 2013). Results are expressed in mean ± SD.

## Results

Among the 392 respondents, 196 were still in training, while 121/54 were working as young specialists/resident-fellow respectively. The length of ICM training programmes was of 4.2 ± 2.6 years; in 45% of cases it was a joint programme with other specialties (mostly anesthesiology and internal medicine). The attended programme did not clearly define competencies for 36% of respondents, whereas bedside teaching and grand rounds represented 54% and 65% of used knowledge and skill teaching methods. Formal resuscitation courses were mandatory in 52% of cases; 70% of formal practical training were funded. Training programmes could be implemented with greater access to courses, scientific events and journals. Independence in taking clinical decision was appropriate in 76% of cases. Average week workload was 53.2 ± 12.4 hours, with 5.6 ± 7.7 night shift per month. Considering the extra workload, neither financial nor time compensation were provided to 60 % and 73 % of respondents respectively. Recipients' monthly salary were different: in 20% of cases, net allocation exceeded 4000 euros, while 30% were paid less than 2000 euros. Workload was evaluated as heavy in 53% of cases (too heavy in 8%) and moderate in 39%; personal-life was rated as good in 27% of cases, fair in 44% and poor in 24%.

## Conclusions

Most ICM training programmes define competencies and training objectives; nevertheless, nor standards of assessment or duration of training are uniform. No speculation can be made on how training is actually affected by different European ICU-staffing systems. Besides, training and working cannot be parted: more than half of respondents defined their workload as heavy and nearly half of them considered their personal-life just fair. Mutual recognition of the speciality need both common training framework and a multidisciplinary ICM core curricula: this would probably create the firm foundation and consistent standard required to train intensivists to a uniform figure across the EU.

## Grant Acknowledgment

This survey was endorsed by ESICM.

